# Temporomandibular joint disc repositioning using bone anchors: an immediate post surgical evaluation by Magnetic Resonance Imaging

**DOI:** 10.1186/1471-2474-11-262

**Published:** 2010-11-12

**Authors:** ShanYong Zhang, XiuMing Liu, XiuJuan Yang, Chi Yang, MinJie Chen, Majd S Haddad, ZhuoZhi Chen

**Affiliations:** 1 Department of Oral and Maxillofacial Surgery, Ninth People's Hospital, School of Medicine, Shanghai Jiao Tong University, No. 639, Zhi Zao Ju Rd, 200011, Shanghai, People's Republic of China; 2 College of Dentistry, University of Iowa, Iowa 52242 USA

## Abstract

**Background:**

Open joint procedures using bone anchors have shown clinical and radiograph good success, but post surgical disc position has not been documented with MRI imaging. We have designed a modified technique of using two bone anchors and 2 sutures to reposition the articular discs. This MRI study evaluates the post surgical success of this technique to reposition and stabilize the TMJ articular discs.

**Methods:**

Consecutive 81 patients with unilateral TMJ internal derangement (ID) (81 TMJs) were treated between December 1, 2003, and December 1, 2006, at the Department of Oral and Maxillofacial Surgery, Ninth Peoples Hospital, Shanghai, Jiao Tong University School of Medicine. All patients were subjected to magnetic resonance imaging before and one to seven days post surgery to determine disc position using the modified bone anchor technique.

**Results:**

Postoperative MRIs (one to seven days) confirm that 77 of 81 joints were identified as excellent results and one joint was considered good for an overall effective rate of 96.3% (78 of 81 joints). Only 3.7% (3 of 81) of the joints were designated as poor results requiring a second open surgery.

**Conclusions:**

This procedure has provided successful repositioning of the articular discs in unilateral TMJ ID at one to seven days post surgery.

## Background

The temporomandibular joint (TMJ) is the only diarthrodial joint of the human jaws. The joint is formed by the bony articulations of the mandibular condyle and the temporal bone (glenoid fossa and articular eminence). Interposed between the condyle and the fossa is a piece of dense, avascular fibrous connective tissue, the TMJ disc. This disc divides the joint into superior and inferior joint compartments, which normally do not communicate with each other. The disc and condyle are in a normal anatomic relationship if the posterior band of the disc is located above the condylar head when the mandibular condyle is centrically positioned in the fossa. Because the bilaminar tissue posterior to the disc is relatively weak, TMJ disorders are a relatively common condition with an estimated incidence rate of 28% ~ 88% [1]. Their most common cause is anterior and/or medial displacement of the disc, also known as TMJ internal derangement (ID), which can cause various degrees of pain and dysfunction. Previously reported clinical results of surgical TMJ disc repositioning procedures have been variable, with failures related to a lack of long-term stability, indicating a need for improved methods of disc stabilization [2]. Since 1990s, the international community has been using arthroscope in the treatment of TMJ disc displacement, which was also tried in our department with an improved clinical efficacy [3-6]. Unfortunately, the technical requirement was relatively high, so it was very difficult for the patients in the late stages of ID, especially those with severe disc deformation or thickening bilaminar tissue. In addition, the suture was connected with soft tissues in the anterior wall of the external auditory canal, which caused difficulties in replacing the disc or instability after its repositioning. To overcome this problem, the disc had to be fixed to hard tissues. Open joint procedures using bone anchors by Mehra and Wolford [7] have shown clinical and radiograph good success, but post surgical disc position has not been documented with MRI imaging. This study presented a surgical technique that used a bone anchor to stabilize the TMJ disc, and to assess the disc position using MRI evaluation.

## Methods

Between Dec 2003 and Dec 2006, 81 consecutive patients (81 joints) diagnosed as ID were treated with the use of the anchor in TMJ articular disc-repositioning surgery. Some patients suffered from bilateral joints disease, but one side did not in accordance with the diagnostic criteria of Wilkes-Bronstein classification for TMJ disorders [8], so these sides were not included in this study. There were 23 men and 58 women, with a mean age of 38.5 years (range 23-74). The mean duration of ID before disc-repositioning was 12.06 months (range 0.5-60). Of all 81 patients (81 joints), 3 patients (3 joints) with whom arthroscopic surgery could not be accomplished, were retreated by open disc-repositioning alternatively. Before operation, written informed consents were obtained from each participants enrolled in the study, and the study was also approved by the university ethics Committee.

All 81 patients (81 joints) were evaluated by clinical examination and MRI, which were in accordance with the diagnostic criteria of Wilkes-Bronstein classification for TMJ disorders [8]. Patients diagnosed as III ~ V stages of ID were included in this study (Table 1). The clinical characteristics of ID mainly contain snapping, pain, jaw dysfunction or movement restriction [8]. The detailed inclusion criteria were as follows: Stage III patients with pain, mild jaw dysfunction or movement restriction, and anterior disc displacement without reduction and mild disc hypertrophy as indicated by the imaging; Stage IV patients with chronic pain, moderate jaw dysfunction or movement restriction with the imaging findings indicating anterior disc displacement without reduction, severe disc hypertrophy and osseous abnormality; Stage V patients with chronic pain, crepitation and severe jaw dysfunction; in this case the imaging findings indicated anterior disc displacement without reduction accompanied by disc perforations, severe disc deformation and degenerative bone changes. The procedure and the MRI evaluation were conducted at the department of Oral and Maxillofacial Surgery, Ninth People's Hospital, Shanghai Jiao Tong University School of Medicine.

**Table 1:** The distributions of various ID stages through disc anchorage

Stage	Cases	Percentage (%)
III	44	54.32
IV	25	30.86
V	12	14.81
Total	81	100.00

TMJ disc anchors, which were self-inserting and non absorbable titanium screw (CBMA 2.0-7-105, CiXi Cibei Mouth Cavity Instrument Co., Ltd.) with a length of 5 mm, were originally developed for use in orthopedic surgery procedures. The head and screw threads transited smoothly, forming a groove which was easy for the anchor suture to tie a knot. A special device was used to insert the anchor in the condyle 2-0 Ethibond suture (ETHIBOND*EXCEL, GREEN BRAIDED Polyester suture, ETHICON, INC), which created advantages such as little rejection, better compatibility and non-absorption. Although its disadvantages included definite irritation and inelasticity, the Ethibond suture was thought to be of low rejection, high intensity and regarded as an ideal suture. It was 75 cm long, with two suturing needles at both ends, so that it could be cut into two equal anchor sutures.

**Figure 1:**
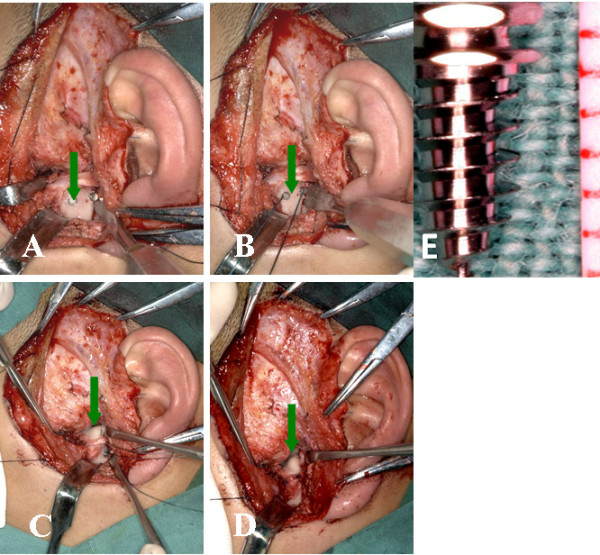
**Titanium anchors and sutures during the procedure**. A showed the titanium anchor in the condyle (green arrow). B showed the sutures tied in the titanium anchor (green arrow). C and D showed the disc repositioned and sutured (green arrow). E showed the actual anchor.

**Figure 2:**
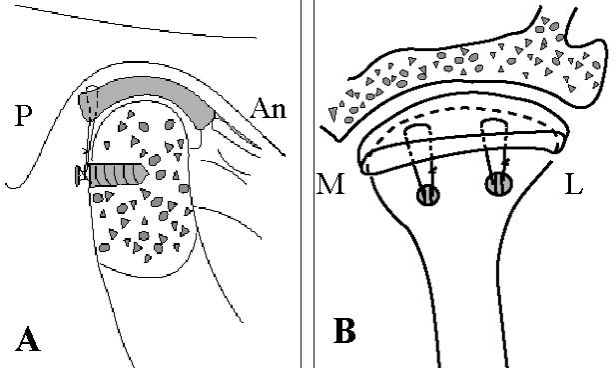
**The titanium anchors and sutures' position**. A. The cross-section of the condyle illustrates the titanium anchor positioned in the posterior cortical bone and the disc repositioning position in the sagittal condyle. P indicates posterior and L indicates anterior; B. Posterior view of the condyle showing the artificial ligaments secured to the posterior band of the repositioned articular disc. Two sutures are passed from the anchor to the disc in horizontal mattress fashion to stabilize the repositioned disc. The sutures tied in the titanium anchors. P. posterior; An. anterior; M. medial; L. lateral

In addition to the pre-operative routine examinations, MRI was also carried out with all patients to determine the disc position, shape and condylar changes. The procedure was carried out in the following sequence: ①The patient was put under general anesthesia through nasal intubation and disinfected routinely. A modified "L" shape incision was used by the authors to gain access to the TMJ area and avoid damaging the facial nerve. The superior and inferior joint spaces were entered, and the disc was identified and mobilized. The disc shape, disc length and condyle were evaluated visually. If the condylar bone spur was present, it had to be repaired during the disc repositioning. ②Anterior release was carried out in the same way as in the arthroscopic anterior release [9]. The anterior, lateral, and sometimes the medial ligamentous attachments were released completely using 11^th ^blade, if indicated, to permit passive repositioning of the disc freely over the condylar head. ③Two TMJ anchors were implanted into the trailing edge of the posterior condylar slope, which was 8 to 10 mm below the top of the condyle just in the middle of lateral-middle junction and medial-middle junction using a standard anchor inserting device. ④After being tied in to two anchors, the 2 Ethibond sutures were then secured to the disc in a horizontal mattress fashion in the junction of the posterior zone and the bilaminar zone. One suture is placed through the medial aspect of the posterior band of the disc, and the other is placed through the lateral aspect of the posterior band. ⑤The assistant pushed the bilaminar zone and the disc to the normal position with the suture strained, and made the patients re-open and close their mouth for two times, to ensure the appropriate position of the condyle fixed 6 to 7 knots. For the partial lateral or medial displaced disc, a single anchor was used near the medial or the lateral in the condyle. ⑥The remaining tissues including the capsule, subcutaneous tissue, and skin were then closed in a routine manner. The position of anchor screws and sutures are shown in Figure 1-2. MRI evaluations were taken to confirm the disc position within one to seven days post surgery.

**Figure 3:**
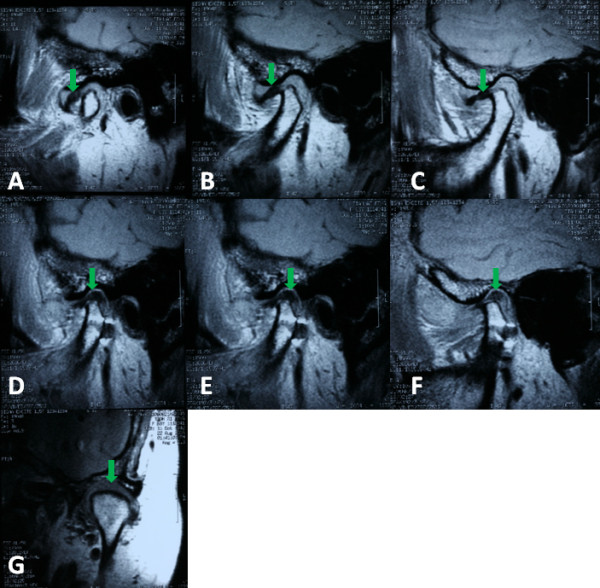
**The disc position on MRI before and after operation**. A, B, C showed the displaced disc anterior to the condyle (green arrow). D, E, F, G showed the displaced disc repositioned in normal position (green arrow)

Pre and postoperative MRI scans were obtained using a 1.5-T imager (Signa, General Electric, Milwaukee, WI) with bilateral 3-inch TMJ surface coil receivers according to the routine sequence [10,11]. Pre- and postoperative MRIs were performed to obtain the evidently repositioned disc, and postoperative MRIs were taken at varying intervals between 1 and 7 days after the operation. The parameters for the sagittal and coronal images were as follows: repetition time (TR), 500 ms; echo time (TE), 25 ms; number of excitations, 2; field of view, 12 cm. A slice thickness of 1 mm with a skip of 0.3 mm and a matrix of 512 × 256 pixels was used. To eliminate any biases, the imaging diagnoses were completed as described by Holmlund [12]. All MRI films were interpreted blindly before the operation by the same TMJ specialist and a radiologist who regularly evaluated the TMJ diseases. They assessed the images separately and made similar evaluations. When their evaluations differed, a third specialist evaluated the images. We also made three levels of 1 cm, 2 cm, 3 cm tongue depressors placed between the upper and lower teeth to stabilize the mandibular position and to achieve the consistent mouth opening, so as to get more accurate comparison of the disc position for the MRI evaluation before and after the operation. For the same patient, three sagittal planes and three coronal planes on MRI films in the same position before and after surgery (Figure 3) were compared under 3 different levels. This evaluation method had proved its effectiveness, based on Zhang SY, et al [13]. The evaluation criteria were as follows: 1) reposition in 3 sagittal parts is excellent, 2) reposition in 2 parts is good, and 3) none or only 1 reposition is poor. Excellent and good evaluations were regarded as successes (if there was disc displacement in only 1 or 2 levels, only replacement of all levels was regarded as a success).

## Results

Post-operative MRIs confirmed that 95.06% of the joints (77/81) were excellent, 1.23% of the joints (1/81) was good, 3.70% of the joints (3/81) were evaluated as poor, in which the disc was not replaced. Cases evaluated as "excellent" and "good", were calculated as successful cases, so the total effective rate was 96.30% (78/81). Only 3.70% of the joints (3/81) were poor. A second open surgery was performed for those 3 patients and satisfactory results were obtained finally. Among those 3 patients, 1 patient (1 joint) was replaced by a temporal myofascial flap and the other 2 patients (2 joints) had a TMJ replacement.

## Discussion

Although Annandale [14] first described surgical disc repositioning of the displaced TMJ disc in 1887, it was not until 1978, when Wilkes [15] used arthrography to describe the anatomy, form and function of the TMJ, that disc repositioning gradually became an accepted surgical technique. Before that time, the routinely recommended treatment for TMJ ID was either to do nothing or to remove the disc. In 1979, McCarty et al [16] repositioned the TMJ disc by a posterior wedge resection (2 mm) of the bilaminar zone, and the success rate was reported to be 94%. However, the similar success with this technique was not achieved by other surgeons. This led to many kinds of new or modified TMJ disc-repositioning surgery with various success rates [17]. Some physicians have applied arthroscopic suturing technique to reposition the disc, however, thus far, there has been no successful report of stable effect [17]. Mehra and Wolford [18] first inserted only one mitek anchor into the condylar process and fixed the disc with special suture in the treatment of 105 patients (188 joints), and achieved a good therapeutic effect. But the effects were only evaluated by clinical examination, without the imaging evaluation of the disc position. In our study, in order to have a stable repositioning of the disc whose diameter was more than 3 cm from medial to lateral, we implanted 2 TMJ anchors for 2-point stabilization of the disc, into the margo-inferior junction in the posterior slope of the condylar process (Figure 2), just in the middle of lateral-middle junction and medial-middle junction, which differed in the study of Mehra and Wolford [17]. Postoperative MRIs confirmed that 96.30% of patients (78/81) were accurately repositioned. Sembronio [19] introduced a similar disc repositioning technique except the absorbable anchor screw. Meanwhile, their postoperative clinical and imaging evaluations were not reported, either.

In the study of Mehra and Wolford [18], patients with a history of less and more than 4 years were compared, and the statistic analysis showed that there was significant difference in the success rate between the two groups. The success rate of the former group was more than 90%, while the later one was only 68%. Mehra and Wolford insisted on early treatment for ID based on the data stated above, which was consistent with our view. Although the patients included were diagnosed as III ~ V stages according to the Wilkes-Bronstein criteria, arthroscopic disc repositioning was first used for the disc without severe deformation. After all, arthroscopic surgery has incomparable advantages superior to open surgery for its minimal invasiveness, which has been widely used in our department with an efficiency rate of about 97% [13]. However, this arthroscopic disc repositioning was not suitable for some patients diagnosed as IV or V stages of ID. In our study, 45.68% of the patients were over IV stage, thus strictly following the indications was very important. Except clinical symptoms, high resolution MRI is of great value for choosing proper patients. Based on the literature [8] and our own experience with MRI evaluation for the disc position, length and shape, as well as the early change of the condylar process and glenoid fossa, we summarized the following MRI indications. ①Although conspicuous disc displacement, degeneration and thickening of the biliminar zone existed, the disc also retains double-concave shape which could not be repositioned easily under arthroscopy. ②No disc intermission with fibrous tissue on sagittal T_1_-weighted MRI.③The anteroposterior diameter of the disc was longer than half of the condyle process on sagittal T_1_-weighted MRI. ④the disc diameter from medial to lateral was larger than that of half of the condyle process on coronal T_1_-weighted MRI. Disc repositioning was carried out in cases of disc perforation in the biliminar zone for the patients in V stage, otherwise, it was excluded.

Delicate surgical procedure is essential for an effective treatment and the following points should be noted: ①Minimize the damage to the facet cartilage and the synovial membrane. ②Anterior release should be dissected completely and the obstacles for disc movement should be removed thoroughly. ③TMJ anchor should be inserted in the inferior border of the condylar posterior bevel rather than in the joint surface to avoid damage to the surface. ④When inserting the anchor screw, the action should be light and soft to prevent splitting the cortical bone and loosening the anchor. ⑤When fixing a tie, the condyle should be on the posterior and the superior of fossa. ⑥The reset direction in the sagittal and the coronal plane should be strictly inspected to make sure that its suture traction direction was exactly the same with the anteroposterior axis of the disc. ⑦Horizontal mattress sutures should be applied from medial to lateral with 2 or 3 sutures, so that the disc is anatomically repositioned and stabilized. ⑧At the end of the surgery, the trailing edge of the disc will take as much as possible on the 11 o'clock (right joints) or 1 o'clock (the left side of the joint) position, which can offset the possible relaxation after a suture knot. ⑨Mouth opening exercises should be taken earlier to promote the recovering of the joint function.

## Conclusion

This technique provides a method to reposition the articular discs confirmed by MRI immediately post surgery. MRIs confirmed that over 96.3% of the patients (78 of 81) had successful disc repositioning at the immediate post surgical time interval, however, long-term follow-up studies are required to validate the success of this treatment approach.

## Competing interests

The authors declare that they have no competing interests.

## Authors' contributions

**SYZ **wrote the paper. **CY **participated in the design of the study. **CY**, **SYZ **and **MJC **carried out the operation, and recorded the patients' data. **XML **performed the statistical analysis and interpretation of data, and drafted the manuscript. **XJY, MJC**, and **ZZC **participated in the analysis and interpretation of data, and reviewed the manuscript. **MSH **participated in the acquisition of data and corrected the English grammar. All authors read and approved the final manuscript.

## Pre-publication history

The pre-publication history for this paper can be accessed here:

http://www.biomedcentral.com/1471-2474/11/262/prepub
